# Non-antibiotic prevention and treatment against *Acinetobacter baumannii* infection: Are vaccines and adjuvants effective strategies?

**DOI:** 10.3389/fmicb.2023.1049917

**Published:** 2023-01-25

**Authors:** Yue Hu, Xianqin Zhang, Shanshan Deng, Changwu Yue, Xu Jia, Yuhong Lyu

**Affiliations:** ^1^Yan'an Key Laboratory of Microbial Drug Innovation and Transformation, School of Basic Medicine, Yan'an University, Yan'An, China; ^2^Non-coding RNA and Drug Discovery Key Laboratory of Sichuan Province, Chengdu Medical College, Chengdu, China; ^3^School of Basic Medical Sciences, Chengdu Medical College, Chengdu, China

**Keywords:** *Acinetobacter baumannii*, whole-cell vaccine, OMV, DNA vaccine, recombinant protein subunit vaccine, adjuvant drugs

## Abstract

*Acinetobacter baumannii* (*A. baumannii*) is a Gram-negative opportunistic pathogen widely attached to the surface of medical instruments, making it one of the most common pathogens of nosocomial infection, and often leading to cross-infection and co-infection. Due to the extensive antibiotic and pan-resistance, *A. baumannii* infection is facing fewer treatment options in the clinic. Therefore, the prevention and treatment of *A. baumannii* infection have become a tricky global problem. The requirement for research and development of the new strategy is urgent. Now, non-antibiotic treatment strategies are urgently needed. This review describes the research on *A. baumannii* vaccines and antibacterial adjuvants, discusses the advantages and disadvantages of different candidate vaccines tested *in vitro* and *in vivo*, especially subunit protein vaccines, and shows the antibacterial efficacy of adjuvant drugs in monotherapy.

## 1. Introduction

*Acinetobacter baumannii* (*A. baumannii*) is a Gram-negative pathogen with strict aerobic and non-lactose fermentation. *A. baumannii* often causes nosocomial and community-acquired infections, such as skin and soft tissue infections, urinary tract infections, meningitis, bacteremia and pneumonia (Morris et al., 2019). *A. baumannii* can produce drought-resistant and disinfectant-insensitive biofilms that enable it to survive in the environment for a long time, and widely exists on biological or non-biological surfaces like medical devices (Harding et al., 2018). Due to the lack of skin barrier protection in the wound, *A. baumannii* pathogens can directly invade the wound, causing blood infection, and the combined infection seriously affects the treatment and prognosis.

Antibiotic abuse led to the accelerated mutation of *A. baumanni* (Gellings et al., 2020). Myxins, polymyxins, and tigecycline are the final treatment strategy. However, the final strategy is invalid for some antibiotic-resistant strains (Holmes et al., 2016). Antibiotic resistance has become one of the world's most urgent public health problems. Therefore, new treatment and control measures are urgently needed. Drug-induced resistance in bacteria is common, and vaccines can effectively reduce the spread and emergency of antimicrobial resistance (AMR). Vaccines target more bacteria than antibiotics, act early, and induce a more robust immune response (Vrancianu et al., 2020). The study on the *A. baumannii* vaccines mainly includes the whole-cell, ghost, OMV, protein subunit, and DNA. We mainly focused on the latest progress in studying protein subunit vaccines against *A. baumannii*. In addition to vaccines, non-antibiotic adjuvant drugs also inhibit the growth and infection of *A. baumannii*, and the effect of monotherapy should not be underestimated.

## 2. Antibiotic resistance

Multidrug-resistance (MDR) means germs gained resistance to three or even more antimicrobial agents; extensively drug-resistant (XDR) pathogens are resistant to all drugs except colistin and tegacycline, while pan-drug-resistant bacteria (PDR) are resistant to all antibiotics even including colistin and tegacycline. Due to the abuse of antibiotics, MDR-, XDR-, and even PDR-*A. baumannii* isolates are ever-growing (Islam et al., 2022). In a survey of drug-resistant phenotypes of *A. baumannii* isolated from clinical samples in northeastern Iran, the rates of the resistant *A. baumannii* isolates against ceftazidime, tobramycin, imipenem, ciprofloxacin was 96.6, 97, 97, and 97.4%, respectively, 74.75 and 73.13% isolates were classified as the MDR and XDR strains (Mirzaei et al., 2020). *A. baumannii* infection is facing the situation that no drug available, over 8,500 hospitalized cases and estimated 700 deaths per year, it is ranked as an urgent threat by World Health Organization (WHO) and Center for Disease Control and Prevention (CDC) (Alamneh et al., 2022).

Antibiotic resistance of *A. baumannii* occurs through a variety of mechanisms, including target mutation; outer membrane permeability reduction that makes it difficult for drugs to enter; multi-drug efflux pumps, for example, the three egress systems of resistance nodulation division (RND): AdeABC, AdeIJK, and AdeFGH; enzymatic drug degradation, like β-lactamases and aminoglycoside-modifying enzymes (Gedefie et al., 2021; Jouybari et al., 2021). Drug-resistant plasmid acquisition is an important reason for the increase of XDR and PDR worldwide. Meanwhile, conjugative plasmid carried in *A. baumannii* containing the resistance determinants plasmid inhibits the VI type secretion system (T6SS), thus reducing bacterial competitors in the host. This advantage benefits *A. baumannii* itself for augmenting (Geisinger and Isberg, 2017). Virulence of *A. baumannii* colonize biofilm, allowing the bacterium to evade the host immunity so that many biofilm-associated infection treatments are ineffective (Sarshar et al., 2021). These mechanisms contribute to resistance to trimethoprim, chloramphenicol, fluoroquinolones, aminoglycosides, macrolides, and β-lactams. Although colistin is deemed the last therapy against MDR *A. baumannii*, the loss of lipopolysaccharide and the increase of lipid A target modification in the pmrAB 2 component system even result in colistin-resistance (Geisinger and Isberg, 2017). These difficulties and problems in therapies have to be overcome by new strategies.

## 3. Vaccine

### 3.1. Whole-cell vaccine

#### 3.1.1. Inactivated whole-cell vaccine (IWC)

IWC is a traditional vaccine. Bacteria lose activity and toxicity by physical heating or chemical treatment with formalin which is safer than attenuated vaccines. Compared with the vaccines composed of a single protein subunit, IWC contains a variety of antigens, so the protective effect is less affected by the down-regulation of membrane protein surface expression caused by antibiotic resistance (Vila et al., 2007). IWC can stimulate the body's response to multiple antigens on cells, maintaining relatively stable immunogenicity induce strong and extensive protection. IWC vaccines have been successfully applied in viral bacteria, such as *Vibrio cholerae, Mycobacterium Bovis, Pertussis, Salmonella typhi*, and *Yersinia pestis* (Moyle, 2015).

Although the IWC vaccines against *A. baumannii* have not been used in clinical practice, they have shown strong protection in various studies. ATCC19606 and clinical strains Ab-154, 113-16 showed complete protection to C57BL/6 mice by active and passive immunization after inactivation (McConnell and Pachon, 2010). More studies based on IWC have evolved, and the enhancing safety and immunogenicity of IWC have appeared. Meritxell et al. found lower tissue bacterial loads and serum levels of pro-inflammatory cytokines interleukin (IL)-1b, tumor necrosis factor (TNF)-α, and IL-6 after immunization with lipopolysaccharide (LPS)-deficient inactivated *A. baumannii* ATCC19606 (IB010). IB010 reduced inflammation and cross-protected mice from LPS-containing ATCC 19606 and Ab-154 attacks (Garcia-Quintanilla et al., 2014). Inactivated LAC-4 vaccine is inoculated by intranasal immunization, triggering specific immunoglobulin A (IgA) reactions and strong IgG1 and IgG2a. Nasal immunization also largely limits the extrapulmonary transmission of the strains (KuoLee et al., 2015). The study has pointed out that the mice induced higher IgG after injection with MDR *A. baumannii*, which had been exposed to imipenem, than non-antibiotic bacteria, and the serum-mediated lysozyme reaction by antibiotic-exposed bacteria *in vitro* is also strong, with a mortality rate of 0–4.4 ± 7.7%. It may be because antibiotic pretreatment can enhance the protein expression of *A. baumannii* and produce a stronger immune response (Shu et al., 2016). Recently, the first study showed the protection of liposomes-encapsulated whole cell antigens (Lip-WCAgs) in infection model, Liposomes owned strong immunoadjuvant potential, Lip-WCAgs induced the higher production of antibody titers, stimulate immunocyte proliferation, the antisera from immunized mice can inhibited the biofilm formation, and 80% mice survived from challenge (Khan et al., 2022). The latest research shows that in addition to formalin inactivation, the relatively innovative radiation inactivation method also shows 80–100% protection ability (Dollery et al., 2021).

#### 3.1.2. Live attenuated vaccine

Unlike IWC, live attenuated vaccines are made by living cells with pathogenicity change or reduction. The highly conserved *murI* gene is an essential gene for cell walls. Knocking out universal gene targets or common components like cell wall is convenient for virulence attenuation in various bacteria, so we do not need to find specific targets in each bacterium (Dollery et al., 2021). Besides, compared with other live attenuated vaccines, the blockage of the cell wall synthesis reduces the risk that attenuated bacteria propagate into the environment. Except for the cell wall, the virulence factor is also a good target, and the *trxA* gene encodes thioredoxin, a virulence factor that makes *A. baumannii* colonize the intestine. Attenuated *A. baumannii* strain without the *trxA* can completely protect mice from infection (Ainsworth et al., 2017).

Although IWC and attenuated live vaccines are easy to prepare and have high protection, whole-cell vaccines still have safety problems such as incomplete inactivation and re-virulence and may cause nonspecific immunity due to various antigens on the cell surface. However, the mutation may decrease the expression of several antigens. In some aspects, the whole-cell vaccine is more guaranteed than the subunit vaccine, which is merely against an antigen because IWC has a wider range of antigens (Vila et al., 2007).

### 3.2. Bacterial ghost (BG)

The bacterial ghost (BG), without the cytoplasmic components of bacteria, like DNA and protein, lacks replicative ability. Thus, BG is relatively safe. Researchers innovatively used chemical methods to prepare the BG of the Ali190 strain. Mice were inoculated by oral administration, subcutaneous, intraperitoneal, and intramuscular injection. The protection rate of oral administration was 67%, and the others were 100%. The inoculation method has an extremely important impact on protection (Sheweita et al., 2019). Unlike a single subunit protein vaccine, ghosts can produce polyclonal antibodies with broad protective effects because they do not destroy surface antigens. Moreover, the BG can be used as an adjuvant to improve immunogenicity (Hajam et al., 2017).

### 3.3. DNA vaccine

A DNA vaccine is called the third-generation vaccine. A DNA vaccine is an engineered expression vector carrying the required immunogen sequence. DNA vaccine directly translates proteins in host cells. These proteins bind to major histocompatibility complex (MHC) I or MHCII molecules and induce humoral and cellular immune responses against various pathogens, including bacteria, fungi, or viruses. In addition, DNA vaccines seem to be safe and produce lasting immunity, so it is more effective in preventing chronic viral infectious diseases (Li et al., 2012).

NlpA is one of the most important antigens in outer membrane vesicles (OMV). The *nlpA* gene was connected with the expression vector to construct PEGFP-C2-*nlpA*, injected this recombinant vector into BALB/c mice (Hashemzehi et al., 2018). The expression of IgG, IgM, and cytokines in mice serum increased. It indicated that PEGFP-C2-*nlpA* induced humoral and cellular immunity. The mice inoculated with pEGFP-C2-*nlpA* were protected from lethal dose infection. All control mice died within 48 h after infection (Hashemzehi et al., 2018).

Outer membrane protein A (OmpA) fragment was inserted into the plasmid vector pBudCE4.1. The pBudCE4.1-*ompA* vaccine also mediates the increase of serum IgM, IgG, IL-2, IL-4, IL-12, and INF-γ levels in mice (Ansari et al., 2019). Lei et al. prepared a DNA vaccine encoding double genes. *ompA* and *pal* double genes were inserted into the pVAX1 plasmid. Pal is a peptidoglycan-associated lipoprotein in the cell envelope and is related to the peptidoglycan layer. The survival rate in the double antigen vaccine group was significantly higher than the single antigen group and the control group, and the double antigen vaccine has a significant protective efficiency and cross-protection (Lei et al., 2019).

### 3.4. OMV and OMC

Outer membrane vesicles (OMV) of *A. baumannii* are lipid bilayer vesicles containing a variety of antigens, such as LPS, nucleic acids, and a variety of proteins. OMV vaccine of New Zealand meningococcal type B has been successfully applied to humans (van den Broek et al., 2019), but there is no OMV available for humans to treat *A. baumannii* infection yet.

McConnell et al. have confirmed that the OMV vaccine prepared from ATCC 19606 strain protect mice from attack by ATCC 19606 strains but also protects mice from challenges from pan-resistant clinical isolates Ab-154 and 113–16. Because OMV contains a variety of proteins, it stimulates the immune system to produce a broad spectrum antigen-specific and high titer of IgG and IgM. Th1 and Th2 are stimulated to produce specific IgG1 and IgG2c, reducing inflammatory factors (McConnell et al., 2011a).

The engineered *E. coli* OMVs (Omp22-OMV) derived from *A. baumannii* ATCC 17978 also had protective effects on septicemia mice. The titer of Omp22-specific antibody in mice immunized with Omp22-OMV was about 100 times higher than in mice immunized with Omp22. The titer of the anti-AbOMV antibody produced by Omp22-OMV was 500 times higher than the Alum + Omp22 group and 20 times higher than wild DH5α OMV (wtOMV), which also indicated that *E. coli* OMVs induced antibody can cross-reacted with *A. baumannii*. The active immunization and antiserum passive immunization of Omp22-OMV provided significant protection against fatal attacks of *A. baumannii* and reduced viral load, and the survival rate was positively correlated with the dose, OMV could be used as an effective platform for the vaccine (Huang et al., 2016a).

LPS is the main content of OMV, and the endotoxin activity of LPS also has a safety risk. IB010 OMV is produced by *A. baumannii* lacking LPS caused by lpxD gene mutation. After immunization, the titers of specific antigens IgG1, IgG2a, and IgM produced by IB010 OMV increased significantly, the bacterial load in the spleen decreased, and the pro-inflammatory cytokines IL-1b and IL-6 decreased, but the protection rate of 10 mg IB010 OMV was slightly lower than same dose ATCC 19606 OMV or IB010 OMV + purified LPS (100%). Increase to 100 mg, and mice were completely protected (Pulido et al., 2020).

For pan-resistant *A. baumannii*, simple antibiotic treatment has been relatively difficult. Anti-omv antibody (AbOMV antibody) assisted in the killing effect of antibiotics against *A. baumannii in vitro*. AbOMV antibody reduced the MIC of quinolones by four times *in vitro*. In sepsis and pneumonia models, the anti-AbOMV antibody also made antibiotics play a more important role in reducing the bacterial load in organs, improving the survival rate, and reducing the infiltration of inflammatory factors. Antibodies may change the conformation of porin and make antibiotics accumulate in cells to a greater extent, AbOMV antibody is a new way for clinical treatment, but there are also limitations. Because strong drug resistance results from various mechanisms, anti-AbOMV antiserum overcomes only one mechanism of antibiotic resistance (i.e., increasing intracellular drug aggregation) and is insufficient to reverse resistance conditions (Huang et al., 2019).

A recent study has found that the different methods for extracting OMV also affect the yield, proteins, and morphology, thus affecting the protection efficiency (Li et al., 2020). The OMV was extracted from bacterial cells directly by three different methods: sOMV released by cells in the culture supernatant, SuOMV (extracting by sucrose), and natural AbOMV (nOMV), the results showed that SuOMV obtained the highest level of protection immunity by strongly expressing specific serum IgG or mucosal sIgA (Li et al., 2020). This study provides valuable ideas and references for future vaccine preparation.

Like OMV, the outer membrane complex (OMC) contains many cell surface proteins. The OMC vaccine's preventive vaccine and antibody therapy have been shown to have excellent protection (McConnell et al., 2011b). The pro-inflammatory cytokines IL-1β, IL-6, and TNF-α, were decreased, and the survival rate of mice challenged with clinical pan-drug-resistant (PDR) strains was increased. The *lpxD* mutant IB010 strain produced LPS-free OMC, and the endotoxin activity was reduced by 10,000 times. The pro-inflammatory factors of mice inoculated with the IB010 strain were lower than wild-type OMC, but the antibody levels were comparable (Pulido et al., 2018).

### 3.5. Subunit vaccine

#### 3.5.1. Protein subunit

The envelope of *A. baumannii* is an important pathogenic and antibiotic resistance factor. Not all glycolipids or proteins in the envelope can be used as targets for subunit vaccines. For recombinant or purified subunit vaccines, conservation and immunogenicity of antigens are important.

**Figure 1:**
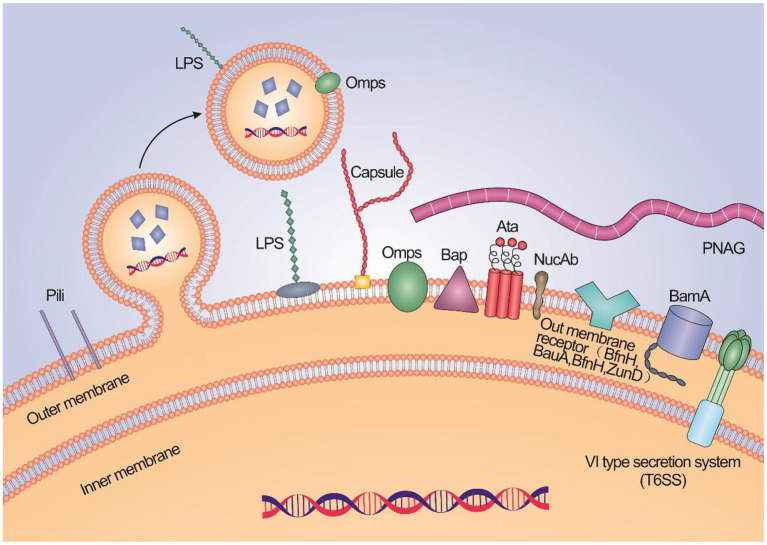
A schematic illustration about the high potential vaccine targets and the typical structure of membrane of *A. baumannii*.

Immunogenic proteins exposed to extracellular or surface are often used as components of candidate vaccines for *A. baumannii*, such as outer membrane proteins (OMPs), porins, channels, receptors, and other functional components (Table 1, Figure 1).

**Table 1:** Subunit protein vaccines.

**Antigens**	**Immune strategy**	**Adjuvant**	**Model**	**Active immunization survival rate (challenge strain)**	**Passive immunization survival (challenge strain)**	**References**
Bap (Kh0060)	IM	CFA, IFA	pneumonia	100% (clinic strain Kh0060)	-	Fattahian et al., 2011
Ata (ATCC17979)	IV	-	pneumonia	-	-	Bentancor et al., 2012b
NucAb (ATCC19606)	IP	CFA	pneumonia	-	30%24h (ATCC19606); 20% 48 h	Garg et al., 2016
FilF (ATCC19606)	SC	CFA, IFA	pneumonia	50% (ATCC19606)	-	Singh et al., 2016
Blp1 (AbIC I, AbIC II)	IM(active), IP(passive)	CFA, IFA	sepsis	60% (AbIC)	100% (AbIC)	Skerniškyt et al., 2019
CsuA/B, FimA (ATCC19606)	SC	CFA, IFA	sepsis	Csu-Fim: 62.5% (ATCC19606), Csu: 37.5%, Fim: 50%	-	Ramezanalizadeh et al., 2020
ABAYE2132 (ATCC19606)	SC	CFA, IFA	peritoneal infection	100% (ATCC19606)	-	Mahmoudi et al., 2020
CAM87009.1 protein (ATCC19606)	IP	CFA, IFA	-	-	-	de Freitas et al., 2021
DcaP, DcaP multiple-epitopes (ATCC19606)	SC	CFA, IFA	respiratory sepsis	2 × LD50 and LD100 DcaP, multiple-epitopes: 100%; 2 × LD100 DcaP/multiple-epitopes:50%/33.3% (ATCC19606)	2 × LD50 DcaP/multiple-epitopes: 66.7%/50% (ATCC19606)	Raoufi et al., 2022
SmpA, PLD (ATCC17978)	SC	Al (OH)3	Sepsis, diabetic	-	SmpA: 33.3~50%, PLD:33.3~66.7% Combined: 50~66.7% (ST191, ST208, ST425)	Li et al., 2017
OmpA (ATCC17978)	SC	Al (OH)3	glycosuria, ketonuria	45–50% (HUMC1)	-	Luo et al., 2012
OmpW (ATCC17978)	IP (active), IV (passive)	Al (OH)3	sepsis	100% (Ab1)	83.3% (Ab1)	Huang et al., 2015
Omp22 (ATCC17978)	SC	Al (OH)3	peritoneal infection	100% (Ab1)	-	Huang et al., 2016a
Oma87 (ATCC19606)	SC	CFA, IFA	peritoneal infection	2 × LD:100%, 7 × LD:25% (ATCC19606)	2 × LD:50% (ATCC19606)	Rasooli et al., 2020
OmpA,Bap,OMV (ATCC19606)	SC	Al (OH)3	sepsis	OMV + Bap: 100% (MDR AB-44 &ATCC19606) OmpA + Bap:100% (ATCC19606)	-	Badmasti et al., 2015
OmpA PKF (ATCC19606)	IP	Al (OH)3	sepsis	PKF:83.3%, OmpA:66.6%, OmpA + PKF:85.71% (ATCC19606)	-	Bolourchi et al., 2019
OmpK/Omp22 (ATCC19606)	SC	CFA, IFA	sepsis	OmpK/Omp22: 66.7%, OmpK: 25%, Omp22: 37.5% (ATCC19606)	-	Guo et al., 2018
CS-PLGA-rOmp22 (ATCC19606)	SC	CFA, IFA	pneumonia	83.33% (ATCC19606), 71.43% (CS-MDR-AB), 66.67% (CRAB), 57.14%(PDR-AB)	-	Du et al., 2021
BauA BfnH (ATCC19606)	SC	CFA, IFA	sepsis	BauA 45%, BfnH40%, BauA + BfnH 43%; BfnH186-41730%, BauA321-635:20%; BfnH186-417+BauA321-635: 33% (ATCC19606)	Anti-BfnH:50%; Anti-BauA+BfnH:50%(ATCC19606)	Aghajani et al., 2019
ZnuD: loop2,5,7,11 (ATCC19606)	SC	CFA, IFA	peritoneal infection	Loop 2: 40%, Loop 5: 30%, Loop 7: 50%, Loop 11: 35%, Loop 2,5,7,11: 100%(ATCC19606)	-	Qamsari et al., 2020
rHyr1p-N (*C. albicans*)	SC	Al (OH)3	Diabetic, pneumonia	60%(HUMC1)	85%(HUMC1)	Uppuluri et al., 2018
OprF25-200 (*P. aeruginosa* ATCC9027)	SC	BCG, Al (OH)3	Albino, peritoneal infection	50% (P. aeruginosa ATCC9027)	-	Bahey-El-Din et al., 2020

CFA, complete Freund's adjuvant; IFA, incomplete Freund's adjuvant; IM, Intramuscular; IP, intraperitoneally; SC, subcutaneously; IV, intravenously.

The biofilm formation on abiotic and biotic surfaces is closely related to the resistance phenotype of *A. baumannii*. So, the conserved regions of proteins associated with biofilm formation by *A. baumannii* can be used as candidate vaccines. Such as biofilm-associated protein (Bap) (Fattahian et al., 2011) and trimeric autotransporter protein (Ata) (Bentancor et al., 2012b), which are involved in infection. Bap is conserved in different clinical strains with the same immunophenotype. Vaccinations of the recombinant Bap protein boosted the significant rise of IgG antibodies, reduced bacterial load, and protected mice from a lethal dose of *A. baumannii* (Fattahian et al., 2011). Ata, binding extracellular matrix/basal membrane (ECM/BM) proteins, Mediating the adhesion and pathogenicity of *A. baumannii* to type IV collagen. Leticia et al. found that antibodies to Ata have the ability of anti-adhesion, sterilization, conditioning phagocytosis, and resistance to *A. baumannii* infection. The passive immunization of anti-Ata serum resulted in significant protection for mice (Bentancor et al., 2012b). Outer membrane nucleases (NucAb) is a protein with nuclease activity on *A. baumannii*, which is related to bacterial pathogenicity and survival in harsh environments. It is non-homologous to human proteins and avoids the possibility of inducing auto-antibodies. It is highly conserved in 40 clinical *A. baumannii* strains (>98%). Recombinant NucAb triggers high antibody titers and inhibits lung inflammation and bacterial loads. Compared with active immunization, the protection rate of passive immunization is relatively high (Garg et al., 2016). Pilus helps bacteria adhere and dissociate on biological or non-biological surfaces. FilF, an uncharacterized putative pilus assembly protein, was predicted by *silico* analysis. The virulence of FilF is still unclear. Subcutaneous injection of FilF, as a candidate conservative protein, significantly reduces the levels of pro-inflammatory factors (IL-6 and TNF-α) and protects 50% of mice from a lethal dose of challenge (Singh et al., 2016). Other proteins associated with biofilm formation and adhesion, such as blp1 (Skerniškyt et al., 2019). Recombinant pilus proteins (CsuA/B and FimA) (Ramezanalizadeh et al., 2020) and fimbrial protein (ABAYE2132) (Mahmoudi et al., 2020) have been shown to have effective protection against *A. baumannii* infection using active and passive immunization. The polyclonal antibody (pAb) produced by recombinant CAM87009.1 antigen derived from *A. baumannii* ATCC 19606, rCAM87009.1 has anti-biofilm activity and against 80% MDR *A. baumannii* strains. After immunization, IgM, IgA, and IgG in the serum of mice are increased, and rCAM87009.1 is a hopeful target (de Freitas et al., 2021). Abundance porin DcaP plays a role in biofilm formation either, it was the first time to report the immune properties of DcaP and multiple-epitope vaccine based on DcaP, a significant increase of the antibody titers of the immunized mice with the multiple-epitope vaccine and DcaP, the relatively high survival rate of the immunized mice with DcaP when received 2 × LD100 of the A. baumannii ATCC 19606 in active immunity, as well as 2 × LD50 bacteria in passive immunity (Raoufi et al., 2022).

Small protein A (SmpA) and phospholipase D (PLD) are virulence factors that help *A. baumannii* spread through blood. Reverse immunology and *in silico* analysis showed that SmpA and PLD might be available candidate vaccines to prevent *A. baumannii* infection. Mice immunized with fusion proteins histidine SmpA and PLD induced humoral immunity with high titer IgG. The content of IL-1β, IL-6, and TNF-a in Broncho-alveolar lavage fluid (BALF) was significantly decreased. Compared with the SmpA and PLD combination, the protection efficiency of single antigen immunization is relatively higher (Li et al., 2017).

The Omps has become one of the most popular candidate vaccines due to its content on the surface of *A. baumannii* and its conservation. For example, OmpA (Luo et al., 2012), OmpW (Huang et al., 2015), Omp22 (Huang et al., 2016b), and Oma87 (Rasooli et al., 2020)have more than 90%, even 99% homology among different strains, and these recombinant proteins have immunogenicity and immunomodulatory properties.

OmpA is one of the most important virulence factors of *A. baumannii*, which is related to adhesion and invasion, apoptosis, biofilm formation, and immune stimulation, and it is involved in OMV a huge amount. OmpA induces polarized IL-4/Type2 responses by increasing immunization doses. High titer IgA is produced by intranasal immunization (Zhang et al., 2016). Immune strategies for combined OmpA vaccines are also evaluated. Farzad used recombinant proteins AbOmpA (8-346aa) and Bap (1-487aa) with different conserved immune dominant regions and OMV (PagL), which reduced LPS endocytosis by expressing 3-O-deacetylase PagL, to immunize disseminated sepsis mice model through separate or combined formulations. In AbOmpA + Bap and Bap plans, Th2 responses were dominant, and Th1 and Th2 both responses were stimulated in the other formulations. AbOmpA + Bap and OMV + Bap induced higher concentrations of IL-4 and IFN-γ, rather than AbOmpA + Bap, OMV + Bap had the best survival rate (Badmasti et al., 2015), accord with expectation, survival curves of combined vaccines were higher than single protein groups (AbOmpA or Bap). Similarly, OmpA was combined with secreted serine protease PKF, but there was no significant difference in survival rates between AbOmpA + PKF plan (85.71%) and the PKF plan (83.3%). Total IgG, IgG2c, and bacterial loads of AbOmpA + PKF even were lower than the PKF plan. This combined vaccine plan did not improve the vaccine efficacy satisfactorily, the PKF plan was more effective, and the combined vaccine plan did not produce a synergistic effect (Bolourchi et al., 2019).

Omp34 is present in more than 1,600 *A. baumannii* strains with homology ≥ 98%, and it has been proven to be an effective candidate vaccine. Considering the insolubility of subunit recombinant vaccines like Omps, the most suitable epitopes on Omp34 for B cells were predicted by bioinformatics and immunoinformatics based on the principle of “antigen minimization” and “high-density epitopes”. “Antigen minimization” means that specific regions of antigenic epitopes can cause a response, and “high epitope density” means that the expression of these epitopes can be increased. After removing the inappropriate regions, a new soluble antigen of Omp34 was designed. This breakthrough method can also be applied to other Omps, But the in silico results are needed to verify by further experiments *in vitro* and *in vivo* (Jahangiri et al., 2018). Similarly, using bioinformatics strategies, Mehdinejadiani et al. identified the linear epitopes of immunoreactive B cells and T cells on OmpA (Mehdinejadiani et al., 2019). Five peptides with the highest score were injected into mice and finally screened. One peptide triggered the immune response. Bioinformatics strategy combined immunization is a valid method to design vaccines (Mehdinejadiani et al., 2019). Due to the bacteria-acquired antibiotic resistance, the target antigens expression may be reduced, which is also a defect of the single subunit vaccine. So, more and more researchers tend to use the multivalent vaccine. Mice immunized with OmpK/Omp22 recombinant fused protein provided significantly greater protection against *A. baumannii* challenge than mice immunized with a single protein alone (Guo et al., 2018). The newest research combined bioinformatics and nanotechnology in vaccine design. Poly(lactic-co-glycolic) acid (PLGA) can deliver antigens, and chitosan (CS) enhances macromolecule permeation on the mucosal surface. PLGA was coated with CS to form CS-PLGA nanoparticles, which could target to deliver antigen to antigen-presenting cells (APC). In this study, the best epitopes of Omp22 were screened by bioinformatics methods, and these T/B cell epitopes were connected in series by 6-aminohexanoic acid, and the multi-epitope polypeptide rOmp22 was chemically synthesized. Finally, rOmp22 was encapsulated by CS-PLGA, forming CS-PLGA-rOmp22 nanoparticles. BALB/c mice were immunized with CS-PLGA-rOmp22, and the levels of immunoglobulin and IFN-γ were higher than uncoated rOmp22. Bacterial load was inhibited, and the protection rate against acute lethal *A.baumannii* infection was 57.14%~83.3% (Du et al., 2021). The other similar research encapsulated Omps with PLGA immunogenic vaccine delivery carrier (OMP-PLGA nanoparticles), OMP-PLGA nanoparticles group generated higher IgG than pure Omps group, The antibody level gradually increased in each immune stage, high level in serum opsonophagocytos is as well as protection for lungs (Gholizadeh et al., 2022). In short, the combination of multi-epitope protein and nanotechnology is a very innovative design direction.

Metal ions are important elements to maintain life and play an important role in the pathogenicity of *A. baumannii*. Metal carriers and receptor proteins are also effective sites for preparing vaccines. For example, outer membrane siderophore receptors, BfnH and BauA, BfnH widely exist in 3,472 *A. baumannii* strains, with more than 97% identity. Immune mice with appropriate fragments containing major B cell epitopes of BfnH, BauA immunogenic regions, and whole protein alone or in combination. Except for no significant difference in reducing bacterial load between the plug of BauA and control, other plans effectively increased IgG titers, reduced bacterial load, and improved survival (Aghajani et al., 2019). ZnuD, a zinc outer membrane receptor, belongs to the TonB-dependent receptor family of iron carriers, and the extracellular loop region of the β-barrel of TonB-dependent receptor protein is an effective immune response region. The bioinformatics server was applied to select the best ZnuD loop to display on LCL. LCL is derived from Neisseria meningitides transferrin binding protein B (TbpB) C flap. There was no protective immune response in septic mice immunized with LCL. When immunized with a single ZnuD loop, 25–50% of mice survived, and mice immunized with mixed four rings were completely immune to infection (Qamsari et al., 2020). This method proves the feasibility of specific residues to prepare vaccines. Similarly, exposed loops (Mirzaei et al., 2020; Vrancianu et al., 2020; Alamneh et al., 2022) of the outer membrane receptor BauA displayed on LCL, among them, loop 7 hybrid antigen and recombinant BauA completely protected mice from infection, and the virtual absence of colonies in lungs, spleen, and liver. BauA loop 7 was as effective as immunization with recombinant BauA (Chaudhuri et al., 2022). This analogous operation is also applied to Omp34 in the latest study, the superior protection of Omp34 loop 3 also proves the hybrid antigen strategy is a viable method (Golestani et al., 2022). The combination of BauA loop 7 and Omp34 loop 3 brought 71.43% protection, higher than alone immunization (Akbari et al., 2022).

Except own recombined proteins from *A. baumannii* can effectively prevent infection, the selected proteins from different genera of bacteria using the principle of homology can also play a protective role. For example, Hyr1 peptides (Hyr1p) from *Candida albicans* (*C. Albicans*) are homologous to OmpA of *A. baumannii*, and both can form mixed biofilms. The anti-Hyr1p antibody can bind to *A. baumannii* instead of proteins with low homology on the *Pseudomonas aeruginosa* (*P. aeruginosa*), which proves the above view we mentioned. The active immunization with recombinant N-terminus (rHyr1p-N) protected 60% of diabetic mice from the lethal dose of *A. baumannii*. The survival rate of passive immunity was 85%. The antibody against peptide 5 of rHyr1p-N also reduced the formation of *A. baumannii*/C. Albicans mixed biofilm *in vitro* and anti-peptide 5 serum had a synergistic effect with antibiotics against *A. baumannii*. Due to the similarity, this antibody can bind to FhaB, OmpA, and outer membrane iron carrier binding proteins on *A. baumannii*. Cross-kingdom antigens vaccine effectively prevents mixed infection by two bacteria in clinics (Uppuluri et al., 2018). At the same, researchers have innovatively detected the potential of the outer membrane protein F (OprF) N-terminal porin domain of *P. aeruginosa* as a vaccine for the first time. OprF 25-200 protein sequence on the N-terminal domain reached 100% identity to OprF from *A. baumannii*. The results showed that this vaccine from *P. aeruginosa* gets cross-reactivity and cross-protection with *A. baumannii*. This vaccine has sufficient immunogenicity to prevent the two pathogens (Bahey-El-Din et al., 2020).

#### 3.5.2. Polysaccharide subunit

Non-proteins on the outer membrane of *A. baumannii*, like polysaccharides, can also be reasonable targets for conditioning or producing antibodies. For example, surface polysaccharide poly-N-acetyl-α-(Holmes et al., 2016; Harding et al., 2018; Morris et al., 2019; Gellings et al., 2020; Vrancianu et al., 2020; Islam et al., 2022)-glucosamine (PNAG) is expressed in many clinical strains. PNAG can potentially be used as a target for active or passive immunization (Bentancor et al., 2012a). K1 capsular polysaccharide has immunogenicity, and anti-K1 capsular antibody MAb13D6 binds to 13% of clinical isolates. K1 capsule can become a potential therapeutic target for passive immunization, but this method is not applicable for K1 negative strains, and the site is limited (Russo et al., 2013). Specific antibodies against capsule polysaccharide (CPS) on clinical isolates SK44 can react with 62% of the strains and even bind to various outer membrane proteins, such as OmpA and Omp38, which are widely present in different strains and have high homology. Therefore, CPS is a feasible therapeutic target (Yang et al., 2017). The type K9 capsular polysaccharide units (various numbers of oligosaccharide repeats) and carrier proteins: bovine serum albumin (BSA), chicken egg albumin (OVA), keyhole limpet hemocyanin (KLH), were synthesized into conjugates by squaric acid chemistry and periodate oxidation. The chemical synthesis remarkably improve the immunogenic properties, and increased secretion of immunoglobulin, particularly IgG, anti-type K9 CPS sera were cross reactive, the TNF-αand IL-10 secreted (Rudenko et al., 2022). Compared to native CPS, synthetic CPS epitopes with simple structures, this character can avoid indefinite immunological results. 16 synthetic oligosaccharides (synthetic glycans) resembling *A. baumannii* ATCC17978 CPS, printed the synthetic glycans on microarray slides. Among them, Tetrasaccharide 20 is screened out by infection sera and a monoclonal antibody (mAb C8), which can be a potential vaccine candidate, but the practical function needs more tests to prove (Sianturi et al., 2022).

#### 3.5.3. New candidates by reverse vaccinology

**Figure 2:**
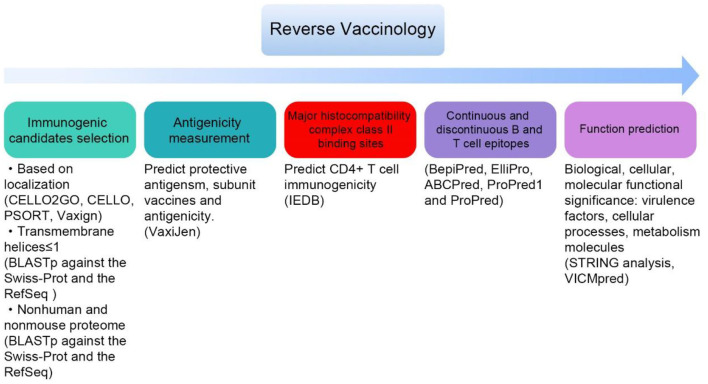
General steps for screening candidate vaccines by reverse vaccinology (some databases and tools in bracket).

Due to its simplicity, short time consumption, and precise and effective cost, reverse vaccinology has become a trend in vaccine design (Figure 2). Candidate proteins can be screened by computer-aided biotechnology from the complete genome and proteome of pathogens in the database, which possesses minimum trans-membrane helices, high adhesion rate, and binding ability to MHC molecule (Shahid et al., 2019). Highly conserved proteins predicted by *in silico* different from human and mouse proteome can achieve the purpose of safety and broad-spectrum immunity (Singh et al., 2022). *A. baumannii* AB030 as the model strain, 13 candidate proteins, including Pal, and MrcB, were predicted (Shahid et al., 2019). Sepideh et al. reported FimA, FilF, acid phosphatase, exported protein, a subtilisin-like serine protease, and three uncharacterized proteins, while they still need to be verified *in vivo* (Fereshteh et al., 2022). Sequential epitope mapping prediction by *in silico* analysis is more sophisticated than candidate proteins screen. Immune-dominant B and T-cell epitopes determined by online tools possess robust protective immune responses. Identified epitopes FPLNDKPGD, FVHAEEAAA, and YVVAGTAAA of the outer membrane protein BamA (Barrel assembly machine protein), fimbrial biogenesis outer membrane usher protein (FimD), and type IV secretion protein (Rhs), respectively, docked with TLR4 receptor to form a multi-epitope vaccine with a high affinity (Ahmad et al., 2019). Two chimeric vaccine constructs linked by seven epitopes filtered from five putative vaccine candidates: APN, mrcB, AdeK, AdeI, and Pal. These constructs have been predicted to have the ability to combat infections caused by *A. baumannii* (Shahid et al., 2021). Multi-antigen vaccines with sequence conservation and strong B-/T helper antigenicity may facilitate a synergistic immune response, especially META-MltDMrdA-OmpA-RpoC combination were suggested (McConnell and Martín-Galiano, 2021). These ideal candidates need further experimental validation.

## 4. Passive immunizing agents

### 4.1. IgY

Passive immunity provides direct and rapid protection. Besides providing immune protection directly from mice serum, the innovative strategy shows infected hens with PDR *A. baumannii* strains and isolated high-purity specific yolk immunoglobulin (IgY) from the yolk. After being injected IgY in the pneumonia mice model, the mortality of mice was significantly reduced from 91.7 to 8.3%. This method is convenient and low cost and can be used for mass production, which is suitable for acute infection. However, the persistence of passive immunization is limited, and serum injection may have the risk of other viral infections (Shi et al., 2017). The murine pneumonia model challenged with LD50 *A. baumannii* before intranasally administered with IgYs against Bap (IgY-Bap), 83% survival was observed, and all control mice died. Bacteria in the lung and spleen of the IgY-Bap group were less than in the control, and biofilm formation decreased. Biofilm disruption increased significantly in IgY-Bap groups (Ranjbar et al., 2022). IgY is a significant strategy for quick and large-scale synthesis since it is more affordable than antiserum IgG and has a similar, equivalent function.

## 5. Adjuvant

Antibiotic resistance and the lack of new therapeutic drugs make the clinical treatment of *A. baumannii* more sophisticated. Besides the vaccine, a non-antibiotic method like adjuvants has also attracted the researchers' attention. These adjuvants have a bactericidal effect, and some have a synergistic effect with antibiotics, which is a promising treatment in clinical practice.

### 5.1. Peptide

Some small peptides have been found to have high bactericidal activity. For example, peptides derived from epithelial cells in humans, human beta-defensins 3 (hBD-3), and human beta-defensins 2 (hBD-2). Multi-drug resistance (MDR) phenotypes *A. baumannii* are particularly sensitive to low concentration hBD-2 (Maisetta et al., 2006; Routsias et al., 2010). Designer antimicrobial peptides A3-APO have obvious treatment for burning or explosive wounds and foot ulcers in various mice models with MDR *A. baumannii* infection, IL-4, IL-6 increased, reduced inflammation and bacterial loads, improve the survival rate, and A3-APO has low toxicity (Ostorhazi et al., 2011a). A3-APO could reduce the bacterial loads by at least two log10 units in the carbapenem-resistant *A. baumannii* infection model, which was more effective than imipenem (Ostorhazi et al., 2011b).

Peptides inhibiting biofilm synthesis have also been proven to have effective antimicrobial properties. 3',5'-cyclic diguanylate acid (c-di-GMP) is the second messenger regulating the growth of the general bacteria; c-di-GMP plays an important role in biofilm formation and has strong immunogenicity. The experiment showed that c-di-GMP had a strong defense against *A. baumannii* and was more applicable to early administration, similar to vaccines, induced chemokines, and neutrophil recruitment (Zhao et al., 2011). Four AMPs peptides prevent biofilm formation, and some of them are synergistic with antibiotics (Gopal et al., 2014). Oral bacteria's DispersinB peptides can disperse and inhibit biofilms. Bacteria became more vulnerable to the broad-spectrum antimicrobial peptide KSL-W when combined with DispersinB peptides (Gawande et al., 2014).

Minimum inhibitory concentration (MIC) values of lead Ceragenin CSA-13 were lower than meropenem, ciprofloxacin, and mycocin, and there was synergy when used together (Pollard et al., 2012; Bozkurt-Guzel et al., 2014). After 20 passages, the MIC values of CSA-13 were less than the MIC values of colistin for *A. baumannii*. CSA-131 has an even stronger ability than CSA-13 (Vila-Farres et al., 2015). Other bacteriostatic agent peptides, such as Hymenochirin-1B, regulate anti-inflammatory cytokines IL-4 and IL-10 (Mechkarska et al., 2013). Mimic peptide apoE and its analog apoE23 have strong antibacterial activity (Wang et al., 2013).

### 5.2. Compounds

2AI-1 comes from the 2-aminoimidazole (2AI) compounds family, targeted biofilm regulator BfmR and is a promising adjuvant for antimicrobial therapy (Thompson et al., 2012). Due to the enhancement of antibiotic resistance, finding new targets for bacteria is particularly important, and the Metal ion transporters are a good target. The Inhibition of the ability to obtain iron can effectively prevent bacteria growth. When serum and transferrin are present, the iron-limiting compound Gallium nitrate significantly inhibits *A. baumannii* growth (de Leseleuc et al., 2012). A synthetic b-aminoketone inhibitor MD3 represents a highly conserved and essential new compound inhibitory target and has a synergistic effect with colistin to enhance the antibacterial ability of both sides (Personne et al., 2014). BAS00127538, a small molecule compound targeting lipid I, can resist MDR *A. baumannii* and enhance its killing effect in combination with colistin (de Leeuw, 2014). Citral, a mixture of monoterpene aldehydes from plants, has no bactericidal effect but a strong detoxification effect with naturally safe annon-toxic, which can reduce the toxicity of *A. baumannii* biofilm by 90%. Citral is targeted to various mechanisms, such as biofilm formation, antibiotic resistance, antioxidant defense, iron acquisition, and type II and type IV secretion systems enhance the sensitivity of *A. baumannii* to the host innate immune system and reactive oxygen species (ROS) (Selvaraj et al., 2020).

### 5.3. Conclusion

The prevention and treatment of *A. baumannii* infection are facing severe challenges. Reverse vaccinology, proteomics, and genomics have also promoted the development of vaccines, providing a good direction and more possibilities for vaccine targets. Although the *A. baumannii* vaccine has achieved good results in animal experiments, it has not been used in clinical practice at present because the antigen targets of the vaccine are relatively single in the current study and cannot achieve comprehensive protection, some proteins are difficult to be purified and low safety. Except selecting targets, the protection rate can be improved by optimizing adjuvants, adjusting doses or inoculation methods, and combining medication. The adjuvant drug results show that it is a promising strategy, but it must be further studied for its toxicological safety *in vitro* and *in vivo* to become a substitute for antibiotics.

## Author contributions

Conceptualization: XJ and YL. Writing original draft: YH. Writing assistance: CY, SD, and XZ. Providing language help: XZ. All authors have read and agreed to the published version of the manuscript.
